# An Electrochemical-Based Point-of-Care Testing Methodology for Uric Acid Measurement

**DOI:** 10.1155/2022/8555842

**Published:** 2022-07-23

**Authors:** Yuetong Zhao, Xia Song

**Affiliations:** ^1^Department of Rheumatology and Immunology, The First Hospital of China Medical University, No.155 Nnajing Street, Heping District, Shenyang 110000, Liaoning Province, China; ^2^Department of Operating Room, The First Hospital of China Medical University, No.155 Nnajing Street, Shenyang 110000, Liaoning Province, China

## Abstract

Point-of-care technology (POCT) is an important method in clinical testing in the future, which can achieve the purpose of rapid analysis. In this work, we assembled an electrochemical POC sensor for uric acid (UA) by surface modification of a screen-printed electrode. Copper nanowires were used as electrode modifiers to achieve high-performance electrochemical oxidation of UA. This electrochemical sensor can achieve linear detection of UA in the range of 10 *μ*M to 2 mM. The detection limit of the sensor was calculated to be 2 *μ*M. Although the detection performance of this sensor is not competitive with high-performance electrochemical sensors, it has been able to meet the needs of POC detection. At the same time, the sensor has excellent anti-interference performance. It has also been used successfully to test urine and serum samples from healthy and gout patients.

## 1. Introduction

The sensitivity, detection speed, and reliability of traditional molecular diagnostic methods have been continuously improved in recent years. However, due to its dependence on the isolation area of environmental control, large biochemical equipment, and the need for strict professional training of operators [1–4], it is still unable to break through barriers and enter scenarios outside the laboratory. POCT, as a new technology, can be directly applied to hospital beds, outpatient clinics, operating rooms, or accident sites [5, 6]. Accompanying diagnosis with just-in-time testing can reduce patient wait times and speed up access to drugs for treatment. At the same time, it is generally automatic analysis from samples to results, so the operator training requirements are low. It can also be performed in hospital laboratory departments, avoiding third-party testing [7–11]. With the rise of connected consumer devices and treatments increasingly tailored to an individual's precise genetics and biomarkers, POCT diagnostics will increasingly enter the daily lives of individual patients and consumers [12–15].

POCT devices are often equipped with biosensors. A biosensor refers to an analytical device for detecting analytes, usually through a small analytical system in which the biological components are fixed to a solid surface and interact with the analyte [16–18]. Electrochemical or optical methods can detect these interactions. Due to the simplicity of the fabrication process, commercially available printed electrodes are an excellent alternative to conventional disk electrodes for inexpensive, high-throughput biochemical analysis [19–21]. Yamaguchi et al. [22] fabricated arrays of micropore-addressable electrodes on commercially available compact discs recordable (CD-R) substrates, and then assembled with microchannel PDMS chips into arrays of electrochemical sensing chips. It was also used for the sensitive detection of serum marker IL-6, with a detection limit of 10 fg/mL. In addition, the group also used a printed carbon electrode array as the substrate, combined with a PDMS microchannel, and inserted a silver reference electrode and platinum wire counter electrode in the channel to form an array electrochemical sensor chip, realizing the simultaneous detection of PSA and IL-6 [23]. Qi et al. [24] combined microfluidic and printed carbon electrode chips to produce an intelligent electrochemical microchip device for rapid and multivariate detection of tumor markers. The device integrates an automated solution delivery unit and a portable electrochemical detection platform, reducing multistep experiments to one-step implementation and interference. At the same time, the sensitive detection of tumor markers AFP and PSA was successfully realized by cyclic voltammetry analysis and the time-current curve method of a multichannel electrochemical instrument.

Uric acid (UA) accounts for about half of the active antioxidant substances in the blood and can change the redox potential or prevent oxidative damage [25, 26]. UA molecules are part of the normal cycle of nucleic acids produced when purines (from DNA or RNA) are oxidized by xanthine oxidase, which is present in peroxidase in most cells. Most mammals deliver uricase in the liver, which breaks down excess UA by oxidizing UA to the highly soluble allantoin and water [27–30]. When individuals develop hyperuricemia due to excessive intake of purines or genetic predisposition, UA saturates body fluids and may precipitate and form sodium urate crystals in joints and other areas, causing gout. UA in the human body has antioxidant properties, and when its concentration is lower than the average level, it will induce some diseases, such as multiple sclerosis, Parkinson's disease, and pernicious anemia [31–33]. Therefore, accurate and rapid detection of UA concentration in the human body plays an important role in treating and diagnosing the above diseases [34–36].

In this work, we constructed an in situ prepared copper nanowire (CuNW)-modified screen printing electrode (SPCE) for UA detection. Firstly, SPCE was used as the substrate electrode, and the copper film was reduced and deposited on the surface of SPCE by the electrochemical method. Then, copper hydroxide nanowires were grown in situ on the surface of SPCE by a mixed solution of ammonium persulfate and sodium hydroxide. CuOHNW was converted to CuNWs by high-temperature heating and electric reduction, and UA was detected by electrochemistry.

## 2. Experiment

### 2.1. Reagents and Instruments

Ammonium persulfate, sodium hydroxide, and sodium acetate were purchased from Sigma-Aldrich. Copper acetate, acetic acid, potassium bicarbonate, D-glucose, ascorbic acid, uric acid, and paracetamol were purchased from China Pharmaceutical Group Co. Ltd. All reagents were analytically pure.

A CHI-660E Electrochemical Workstation (Shanghai Chenhua Instrument Co. Ltd.) has been used for electrochemical experiments. A three-electrode system was used in the experiment, with a screen-printed electrode as the working electrode, a platinum plate as the counter electrode, and Ag/AgCl (saturated KCl) as the reference electrode. The morphology of the samples was observed by a scanning electron microscope (SEM, S-4800, Hitachi, Japan).

### 2.2. Preparation of CuNW/SPCE

The preparation of CuNW was adopted from Xu et al. [37] with some modifications. Firstly, SPCE was placed as the working electrode in the electrolyte of 0.1 M NaAc and 0.02 M Cu(Ac)_2_. The pH of the electrolyte was adjusted to 5.5 by HAc, and the copper film was deposited on the surface of SPCE for 8 min at the constant potential of −0.9 V. The electrodes were then removed from the electrolyte, rinsed with water, and blow-dried with nitrogen. The solution was stirred and kept at room temperature during the entire electrodeposition process. Then, Cu(OH)_2_NW was grown in situ on the surface of Cu/SPCE by soaking the prepared Cu/SPCE in a mixture of 0.133 M (NH_4_)_2_S_2_O_8_ and 2.667 M NaOH for 5 min. SPCE modified with Cu(OH)_2_NW was converted into copper oxide nanowire (CuONW) by heating it at 150°C for 12 h. Then, the SPCE modified with CuONW was used as the working electrode. The CuNW grown in situ on the SPCE was obtained by electric reduction at −0.7 V for 30 min in a 0.1 M KHCO_3_ solution (denoted as CuNW/SPCE).

## 3. Results and Discussion

**Figure 1:**
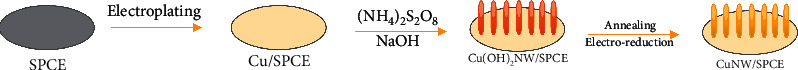
Schematic diagram of the preparation of CuNW/SPCE for UA detection.

In this work, CuNWs were prepared in situ on SPCE. CuNW-modified SPCE was characterized by scanning electron microscopy and electrochemistry, and the electrocatalytic activity of the electrode on the UA was discussed.  Figure 1 shows the schematic diagram of the preparation of CuNW/SPCE for UA detection.

**Figure 2:**
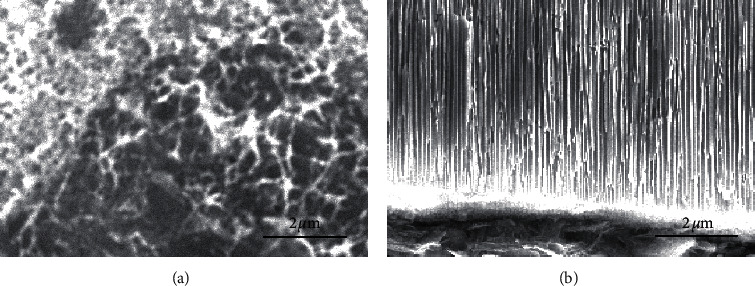
SEM images of (a) SPCE and (b) CuNW/SPCE.

In this work, we proposed a simple method for in situ preparation of CuNW on the surface of SPCE in the liquid phase and characterized the surface morphology by SEM. The rough surface structure of SPCE can be seen in Figure 2(a). Then, Cu(II) was reduced and deposited on the surface of SPCE by electrochemical deposition to obtain Cu/SPCE. Since Cu(OH)_2_ can be converted to CuO as a precursor [38, 39]. It is necessary to prepare Cu(OH)_2_NW in situ on Cu/SPCE surface. Cu(OH)_2_NW was transformed into CuONW by heat treatment in an air atmosphere. Electrochemical reduction of CuONW was carried out in KHCO_3_ electrolyte containing saturated N_2_. As shown in Figure 2(b), free-growing CuNW was formed on the surface of SPCE.

**Figure 3:**
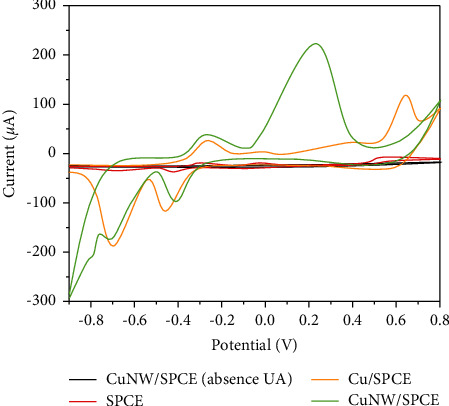
CVs of bare SPCE, Cu/SPCE, and CuNW/SPCE towards 2 mM UA (0.1 M NaOH, scan rate 25 mV/s).

In order to evaluate the analytical applicability of the CuNW/SPCE, the electrochemical performance of the sensor for UA oxidation was investigated in buffer solution. The sensors were placed in buffer solutions with and without UA, and cyclic voltammetry (CV) scanning was performed in the potential range of −1.0 V to 0.8 V, as shown in Figure 3. Bare SPCE has no current response in 0.1 M NaOH solution. This phenomenon indicates that SPCE has no catalytic activity for the electro-oxidation of UA in the applied potential window. The CV curve of CuNW grown on the surface of SPCE is similar to that of Cu/SPCE. These results indicate the formation of CuNW on SPCE [40]. In CuNW/SPCE, the peak at 0.3 V may be due to the formation of soluble material by CuNW and OH^−^. This peak is usually used to study the electro-oxidation mechanism of UA. Compared with the CV curve without UA, some changes occur in the CV curve of the sensor in the presence of 2 mM UA. The peak of the curve disappears at 0.3 V, mainly due to the adsorption of UA onto the surface of CuNW. The peak centered at 0.6 V is the response of the sensor to the electrocatalytic oxidation of UA. A weaker oxidation peak was also observed at 0.6 V for Cu/SPCE than CuNW/SPCE. This phenomenon indicates that CuNW indeed causes the electrocatalytic oxidation signal of UA. In addition, this indicates that the sensor has a high electrocatalytic activity for electrochemical oxidation of UA [40–42].

**Figure 4:**
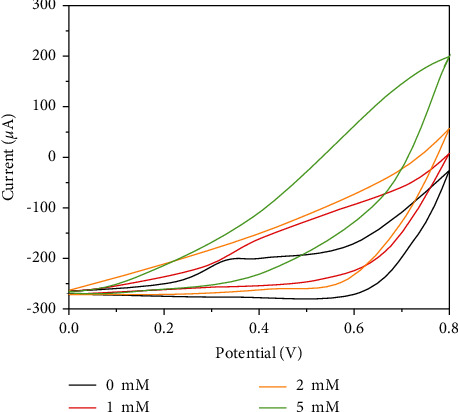
CV of CuNW/SPCE for 0–5 mM UA in 0.1 M NaOH with a scan rate of 25 mV/s.

At the same time, the CV curves of the sensor for different concentrations of UA in the potential range of 0.0–0.8 V were also studied. As can be seen from Figure 4, the catalytic current is generated within the potential range of 0.4 V–0.8 V, and the oxidation peak current increases with the increase of UA concentration. This indicates that the oxidation of UA by CuNW has obvious electrocatalytic behavior [43, 44]. The influence of oxygen on the analytical performance of the sensor was also studied. The UA was scanned with the sensor before and after deoxygenation [45]. The experimental results show that the sensor is not affected by oxygen in UA detection.

**Figure 5:**
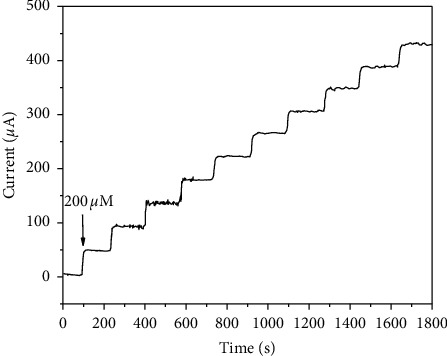
Amperometric responses of CuNW/SPCE upon the successive addition of UA at 0.6 V (0.1 M NaOH).

The current response of the sensor was obtained by adding UA at 0.6 V constant potential. As can be seen from Figure 5, with the continuous dripping of UA, the current response increases step by step and reaches stability within 2 s. The linear response range of UA concentration from 10 *μ*M to 2 mM can be obtained. The linear regression equation is ImA = 2.1 + 220C(mM), and the correlation coefficient *r* = 0.997. The detection limit can be calculated to be 2 *μ*M.  Table 1 shows the comparison of the proposed uric acid sensor with previous reports.

The loss of only 5% of the current response of the CuNW/SPCE to UA was observed in an *N*_2_ atmosphere for one month, indicating good stability of the sensor. The relative standard deviation of 6 measurements of UA with different sensors was used to evaluate the reproducibility of the measurements. The relative standard deviation of 2 mM UA is 4.3%. The results show that UA detection with sensors has high reproducibility.

**Figure 6:**
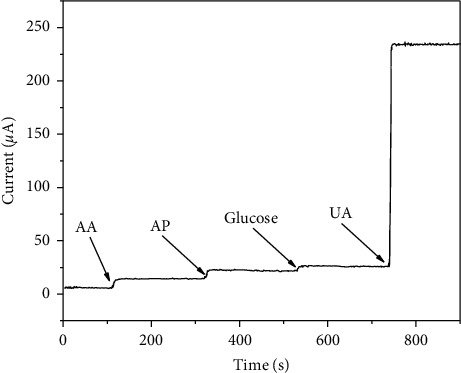
Amperometric response recorded using CuNW/SPCE towards UA and interference species (0.1 M NaOH).

Selectivity is one of the important characteristics of electrochemical UA sensors, especially for detecting UA in practical samples. Ascorbic acid, glucose, and acetaminophen commonly coexist with UA in serum samples as general interfering compounds [51]. Because these small molecule compounds are easily electro-oxidized, they will produce a disturbing signal. Therefore, it is very important to study the influence of these compounds on sensor detection performance. To minimize the interference caused by the presence of anions in the biological medium, naphthol was dropped onto the surface of CuNW/SPCE and the resulting electrode was dried at room temperature for 15 min. The current response of 0.1 mM of ascorbic acid, 0.3 mM of glucose, 0.1 mM of acetaminophen, and 1 mM of UA at 0.6 V was measured. Compared with bare SPCE, these interferences have almost no effect on UA detected by the sensor, as shown in Figure 6. This indicates that the sensor used for UA detection is highly selective.

**Figure 7:**
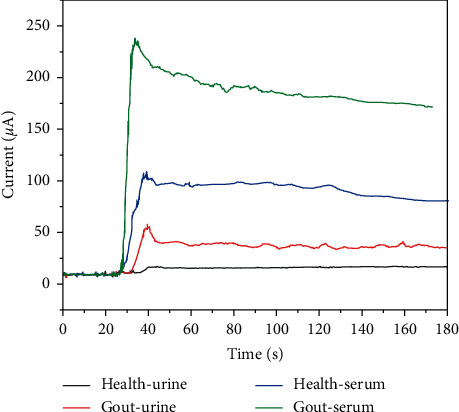
Amperometric response recorded using CuNW/SPCE towards urine samples and serum samples from healthy person and gout patient.

To evaluate the practical application of the sensor, it was used to detect UA in serum and urine samples from gout patients and healthy persons, respectively. The sample was dropped into 0.1 M NaOH solution, the solution was stirred, and the current response at 0.6 V potential was recorded. As can be seen from Figure 7, serum samples of healthy persons and urine samples of gout patients have an obvious current response. When urine samples from healthy people were used, almost no current response was observed.

## 4. Conclusion

In conclusion, we proposed an electrochemical-based POC sensor for rapid UA detection. Commercial SPCE modified by CuNW has excellent electrocatalytic oxidation performance towards UA. This disposable sensor can be used for rapid detection of UA. The sensor provides linear detection of UA in the concentration range of 10 *μ*M to 2 mM, with a detection limit of 2 *μ*M. In addition, this POC sensor has been successfully applied for the detection of UA in urine samples and serum samples.

## Figures and Tables

**Table 1:** Comparison of CuNW/SPCE with previously reported uric acid sensors.

Sensor	Linear range	Lod	Reference
Zn-Al-LDH-QM/MWCNT/CPE	0.1 to 100 *μ*M	0.05 *μ*M	[46]
GNPs@CDs/erGO/GCE	0.1 to 20 *μ*M	0.0335	[47]
PANI/GN	1.5 to 890 *μ*M	0.57 *μ*M	[48]
CDs/GCE	3 to 28.5 *μ*M	0.011 *μ*M	[49]
Graphite modified by fe_3_O_4_@au-cys/pani	20 *μ*M to 1 mM	1.8 *μ*M	[50]
CuNW/SPCE	10 *μ*M to 2 mM	2.0 *μ*M	This work

## Data Availability

The data used to support the findings of this study are available from the corresponding author upon request.
